# Older adults with dementia: knowledge and attitudes of physicians in health units

**DOI:** 10.11606/s1518-8787.2020054002451

**Published:** 2020-11-27

**Authors:** Ana Beatriz Quintes Steiner, Alessandro Ferrari Jacinto, Vanessa de Albuquerque Citero

**Affiliations:** I Universidade Federal de São Paulo Escola Paulista de Medicina Programa de Pós-Graduação em Psiquiatria e Psicologia Médica São PauloSP Brasil Universidade Federal de São Paulo. Escola Paulista de Medicina. Programa de Pós-Graduação em Psiquiatria e Psicologia Médica. São Paulo, SP, Brasil; II Universidade Federal de São Paulo Escola Paulista de Medicina Departamento de Medicina São PauloSP Brasil Universidade Federal de São Paulo. Escola Paulista de Medicina. Departamento de Medicina. São Paulo, SP, Brasil; III Universidade Federal de São Paulo Escola Paulista de Medicina Departamento de Psiquiatria São PauloSP Brasil Universidade Federal de São Paulo. Escola Paulista de Medicina. Departamento de Psiquiatria. São Paulo, SP, Brasil

**Keywords:** Physicians, Primary Care, Health Knowledge, Attitudes, Practice, Patients, Aged, Dementia

## Abstract

**OBJECTIVE:**

To describe the knowledge and attitudes of general practitioners of the basic health network of the city of São Paulo in relation to patients with dementia and identify patterns of attitudes.

**METHODS:**

A total of 10% of the basic health units in the city of São Paulo (n = 45) were randomly distributed into six regional health coordination centers. Up to two general practitioners were interviewed in each unit, with a total of 81 physicians interviewed. They answered the translated and cross-culturally adapted version for Brazil of two British questionnaires, the knowledge quiz (knowledge about dementias) and the attitude quiz (attitude towards the patient afflicted with dementia), as well as a sociodemographic and occupational questionnaire to understand the profile of general practitioners working in primary care. Descriptive data analysis, factor analysis of the main components of the attitude quiz and study of association between attitudes and knowledge were performed, in addition to the multiple linear regression test to determine the relationship between occupational profile and knowledge about attitude patterns in dementia.

**RESULTS:**

The physicians interviewed had a median of five-year graduation time; 35.8% worked exclusively with primary care, and less than 40% had completed, or were attending, medical residency or specialization. Physicians showed a lower knowledge about the diagnosis of dementia than about the epidemiology of the disease and its therapeutic management. Their attitudes towards patients afflicted with dementia resulted in four factors: proactive optimism, delegated optimism, implicit dismay, and explicit dismay. The regression study showed that the attitude of explicit dismay decreases the longer the weekly working hours of the physician in the units, and that the delegated optimistic attitude of the physician decreases in the same situation.

**CONCLUSION:**

Investment in training is essential to improve physicians' performance in the field of dementia in primary care.

## INTRODUCTION

Dementia has an important impact on individuals, on their family, on public health, on society and on the economy ^1^^,^^2^ . Early identification of individuals at the beginning of dementia may be a way to intervene in the progression of the disease, and general practitioners of basic health units (BHU) should be prepared for this reality.

The term “general practitioner” in Brazil is often used indistinctly. According to Cremesp (2010) ^3^ , the term should only be addressed to physicians graduated from the medical school who did not attend medical residency or specialization recognized by the Brazilian Medical Association.

To analyze the attitudes of general practitioners towards patients afflicted with dementia is important to obtain information about the treatment these patients receive in primary care. To do so, it is necessary to understand the level of knowledge of physicians about the subject and what factors influence their attitudes. The aim of this study is to assess the relationship between patterns of attitudes of general practitioners towards older patients with dementia and their level of knowledge.

## METHODS

This study was approved by the Ethics Committee of the Escola Paulista de Medicina (no. 1,113,179) and by the Ethics Committee of the Municipal Secretariat of the City of São Paulo (no. 1,076,944).

A descriptive cross-sectional study was conducted. Data collection occurred in 2017, when the city of São Paulo had 451 BHU, distributed into six regional health coordination centers (2019) ^4^ (North = 88, South = 121, Downtown = 9, West = 29, East = 112, and Southeast = 92).

We estimate that each BHU has two general practitioners. Thus, for data collection, 10% of the BHU in the city of São Paulo (N = 45) were randomly selected, composing a random sample, and 10% of the BHU in the city of São Paulo (N = 45), distributed proportionally into the six regions of the city (North = 9, South = 12, Downtown = 1, West = 3, East = 11 and Southeast = 9). Two physicians were interviewed, when possible, by BHU, totaling an estimated sample of 90 physicians. In case the BHU drawn had only one physician, another BHU from the same region was randomly drawn to complete the sampling. Thus, 48 BHU ( Figure 1 ) were approached, and 81 physicians agreed to participate in the study. The inclusion criterion of the physician was to work in the BHU as a generalist, regardless of training, without additional exclusion criteria.

**Figure:**
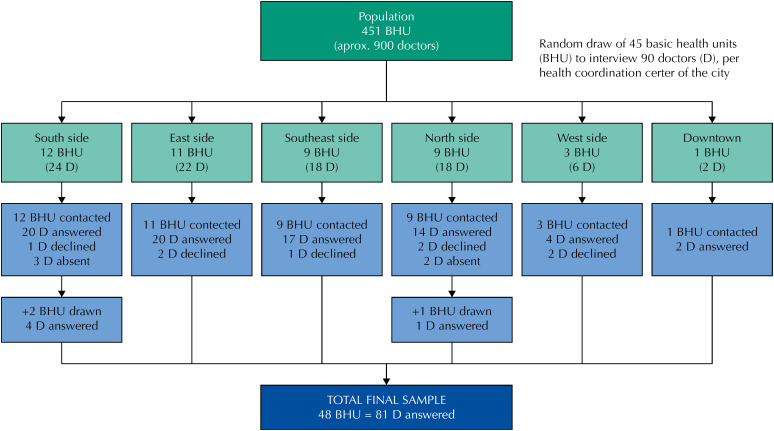
Flowchart for data collection.

Once the units were drawn, the interviewer contacted the health coordination centers of each region. With the permission and release of the survey by the coordinators, the managers of each BHU were contacted by phone or e-mail. Initially, the interviews for the application of the questionnaires were scheduled according to the best day and time for the unit. The interviewer went personally to 34 BHU. At the time of the visits, the two physicians from each unit, after completing the free and informed consent form, answered the questionnaires by themselves. In the remaining units (14), at the request of the BHU managers, the survey was sent by e-mail. Once answered, the surveys were sent back to the researcher. No differences were observed in the quality of the completion of the questionnaires between those who were handed out personally and those delivered by e-mail.

The translated and cross-culturally adapted version of two questionnaires was applied, one about the physician's knowledge of dementia and the other about his attitude towards these patients ^1^^,^^5^ , as well as the sociodemographic and professional questionnaire of the physician, developed for this study.

- Knowledge quiz: translated and cross-culturally adapted for Brazil about general practitioner's knowledge about cognitive alterations ^5^ . There are 14 multiple choice tests, with five possible alternatives, being one of the alternatives “I do not know,” with only one correct answer. The questionnaire is divided into three domains, which cover knowledge related to epidemiological factors (4 questions), diagnosis (7) and management of patients with dementia (3). The questionnaire has been applied to UK general practitioners for some time ^1^ .
- Attitude quiz: translated and cross-culturally adapted to Brazil about attitude towards the patients afflicted with dementia ^5^ . The factor analysis of the instrument in English was coincident with that of the document in Portuguese. There are ten statements related to attitudes and opinions of general practitioners regarding the management and prognosis of dementias. Using a five-point Likert scale, which ranges from “strongly agree” to “strongly disagree,” general practitioners describe how they deal with the care of patients with dementia. In the British version ^1^ , the ten statements compose two attitude factors, one more positive and the other more negative, in the management of the doctor with older adults who have the disease. The analysis of this instrument implied the conversion of the score of each item to the variation from 0 to 100, and considered the reversal of the responses of statements 6, 7, 8, 9 and 10.
- Sociodemographic and occupational questionnaire of the physician: information such as age of the physician, time of training, workload in primary care, having performed medical residency/specialization and having participated in courses on cognitive alterations are important for the description of the sample.

At first, the data were analyzed descriptively. For categorical variables, absolute and relative frequencies were presented, and for numerical variables, summary measures (mean, quartiles, minimum, maximum and standard deviation).

The existence of associations between two categorical variables was verified using the chi-square test, or alternatively, in cases of small samples, Fisher's exact test. If there were differences in distribution, the standardized adjusted residual was used to identify local differences — quads with absolute values above 1.96 show (local) associations between the categories related to these quads.

The linear association between two numerical variables was assessed via Pearson's (r^P^) or Spearman's (r^S^) correlation.

Student's t-test and variance analysis (ANOVA) were used to compare the means between two and three groups, respectively. Both present as assumptions the normality in the distribution of the data and homoscedasticity, which were verified by the Kolmogorov-Smirnov test and the Levene's test, respectively. In case of violation of the homoscedasticity assumption, the degrees of freedom of statistics were corrected using the Brown-Forsythe correction. In case of violation of the assumption of normality in ANOVA, the kruskal-wallis nonparametric test was used alternatively. The mean knowledge scores were compared by Friedman nonparametric test. With differences in means in ANOVA, the distinct groups were identified by Duncan's test to maintain the overall level of significance. For the Kruskal-Wallis test or Friedman test, Dunn-Bonferroni multiple comparisons were used.

To assess the dimensionality of the attitude scale composed of ten items, exploratory factor analysis (EFA) was performed by using principal components and varimax orthogonal rotation. The criterion for selecting the number of factors was eigenvalues above 1. The adequacy coefficient of the Kaiser-Meyer-Olkin (KMO) sample and the Bartlett scouting test were presented, which assesses the overall significance of all correlations between the scale items considered. Then, Cronbach's alpha coefficients were estimated to assess the internal consistency of the items that comprised each factor.

To assess the effects of the training and professional characteristics of physicians, as well as knowledge and attitude scores, the multiple linear regression model was used, which also presents normality in the data as one of the assumptions. Initially, all significant explanatory variables to 20% in the univariate analysis were included in the model. Then the variables not significant to 5% were excluded one by one, in order of significance (backward method).

We adopted the significance level of 5% for all statistical tests. The analyses were performed using the statistical package SPSS 20.0.

## RESULTS

We found that 54.3% of the physicians were men, from Greater São Paulo, 35.8% worked exclusively in the city of São Paulo, in primary care, and 98.8% worked as general practitioners in the BHU. Additionally, 54.3% reported having acquired, during medical school, a good basis for cognitive alterations (dementia) in older adults, and 18.5%, at some point in their lives, had specific courses on the disease. In addition, 25.9% have already undergone medical residency or equivalent specialization, and 19.8% had a strict sense graduation.

On average, physicians had 10 years of medical school (SD = 11.8 years), and a minimum time of 1 year and maximum of 45 years was observed. Regarding the workload in the BHU, the physicians worked, on average, 31.7 hours (SD = 12.3 hours).

The average knowledge per domain was not similar to each other(p < 0.001). On the other hand, the means of epidemiological knowledge and management were similar, and both higher than those of diagnosis.

We found differences in the means of the knowledge score only by taking, at some time in life, courses on cognitive alteration in older adults (p < 0.001); that is, those who took courses had a higher average of total knowledge than those who did not take a course. This effect was maintained when only knowledge in relation to epidemiology was assessed (p = 0.027), but not when knowledge regarding diagnosis was assessed.

A positive correlation was observed between the workload performed at the BHU and the total knowledge score (r^S^ = 0.244, p = 0.028) and diagnosis (r^S^ = 0.295, p = 0.007). There was also a weak positive correlation between time of graduation and management score (r^S^ = 0.237, p = 0.033). The correlation between the percentage of older people seen in the health unit and the knowledge score of the diagnosis was negative (r^S^ = −0.319, p = 0.021).

Exploratory factor analysis was performed to assess the dimensionality of the attitude scale suggested by the data ( Table 1 ). Four factors were obtained, which together explain 63.2% of the total variability of the ten items. The results of factor analysis can be interpreted by “factor loadings,” indicating the items that make up each factor. When observing the set of item by factor, the following qualities were highlighted:

- Factor 1 – proactive optimistic attitude: high values of this indicator show the perception of physicians that they can contribute to improving the quality of care for older adults with dementia and that they are willing to give this care;
- Factor 2 – optimistic delegating attitude: high values of this indicator show that physicians have the perception that the management of older adults with dementia can be done with optimism, but they are not responsible for it and delegate to other instances;
- Factor 3 – attitude of implicit dismay: high values of this indicator show that physicians do not perceive themselves as pessimistic in the care of older adults, but deposit in the family the difficulty in seeing a future in the management of older adults with dementia, as if they were dismayed by proxy;
- Factor 4 – attitude of explicit dismay: high values of this indicator show that physicians have a clear perception that any management of them with older adults with dementia will be unsuccessful.

**Table 1:** Factor loadings, eigenvalues, percentage of explained variance and Cronbach's alpha coefficient of the four factors of the scale.

Items	Attitudes				Communalities *
	FACTOR 1 Proactive Optimism	FACTOR 2 Delegating optimism	FACTOR 3 Implicit dismay	FACTOR 4 Explicit dismay	
3. Much can be done to improve the quality of life of people with dementia	0.756	0.123	−0.100	−0.190	0.632
1. Much can be done to improve the quality of life of caregivers of people with dementia	0.718	−0.039	−0.375	0.154	0.682
4. Providing diagnosis is usually more useful than harmful	0.701	0.091	0.228	−0.004	0.552
5. Dementia is better diagnosed in specialized services	0.218	0.717	−0.285	−0.025	0.643
10. The primary care team plays a very limited role in the care of people with dementia	0.058	0.684	0.181	0.076	0.509
7. It is better to talk to the patient using euphemisms	−0.146	0.604	0.410	0.129	0.572
9. It is not worth referring families to specialized services when they do not want to use them	−0.164	0.207	0.732	0.100	0.616
2. Families prefer to be informed about their relatives' dementia as soon as possible	0.499	−0.147	0.647	−0.081	0.696
6. Patients with dementia may deplete resources with little positive results	0.144	−0.054	0.060	0.892	0.823
8. Treating dementia is often more frustrating than rewarding	−0.293	0.250	−0.001	0.671	0.599
Eigenvalues	2.03	1.50	1.44	1.35	
Percentage (%) of the total variance explained	20.34	15.01	14.43	13.46	
Cumulative percentage (%) of the total variance explained	20.34	35.35	49.78	63.24	
Cronbach's Alpha	0.629	0.468	0.255	0.470	

KMO = 0.534; Bartlett's test for sphericity (45) = 119.47 (p < 0.001).

* Factor analysis allows the decomposition of the variance of each item into two parts: common part and specific part. The part of the common variance – due to common factors – is called communality. Values below 0.50 indicate that the corresponding items are underrepresented in factor analysis.

For all factors, internal consistencies were unacceptable according to Cronbach's alpha coefficients, except for the proactive ^a^ optimistic attitude factor, which proved to be adequate (Cronbach's alpha = 0.629). The scores of each factor were generated as the sum of the values assigned to each item in the reversed form and re-staggered in such a way so they varied from 0 to 100.

No differences were found in the means of the proactive optimistic attitude score, the optimistic delegating attitude and the attitude of explicit dismay in relation to sociodemographic and professional characteristics. However, regarding the attitude of implicit dismay, there were differences in the means of the score of “have a good basis on cognitive alterations in older adults during medical school” (p = 0.010). Thus, those who indicated having received a good basis on the disease in medical school presented a mean attitude score of implicit dismay higher than those who mentioned not having had a good basis.

A negative correlation was found between workload and proactive optimistic attitude score (r^P^ = −0.375, p = 0.001) and explicit dismay (r^P^ = −0.331, p = 0.003). On the other hand, the correlation between the percentage of older people seen at the BHU and the implicit dismay attitude score was positive (r^P^ = 0.277, p = 0.046), but no correlation was found between these data and the optimistic delegating attitude.

Negative correlations were found between optimistic delegating attitude and total knowledge (r^S^ = −0.225, p = 0.044) and diagnosis (r^S^ = −0.305, p = 0.006) score. Additionally, positive correlations were found between attitude of implicit dismay and total knowledge (r^S^ = 0.257, p= 0.021), epidemiological (r^S^ = 0.224, p = 0.044) and management (r^S^ = 0.341, p = 0.002) score.

To assess the effects of the professional characteristics of physicians, as well as knowledge and attitude scores, multiple linear regression models were adjusted for the scores of optimistic delegating attitude ( Table 2 ) and explicit dismay ( Table 3 ). Models were not adjusted for proactive optimistic attitude, because it was not associated with any of the characteristics of physicians and knowledge scores. Regarding the attitude of implicit dismay, models were not adjusted due to low internal consistency (α = 0.255).

**Table 2:** Multiple linear regression model for optimistic delegating attitude.

		Initial model		Final model	
		Coefficient (95%CI)	p	Coefficient (95%CI)	p
Knowledge score				
	Epidemiological	−0.04 (−0.19 – 0.12)	0.628	–	–
	Diagnosis	−0.24 (−0.50 – 0.02)	0.075	−0.25 (−0.49 – −0.02)	0.037
	Treatment	0.03 (−0.10 – 0.15)	0.665	–	–
Workload (hours/week)		−0.51 (−0.87 – −0.16)	0.005	−0.53 (−0.86 – −0.21)	0.002
Attended residency/specialization (ref. = no)					
	Completed	−9.37 (−18.75 – 0.01)	0.050	−9.52 (−18.24 – −0.81)	0.033
	Studying	1.18 (−11.38 – 13.74)	0.852	–	–
Took, at some time in life, courses on cognitive alteration in older adults		−3.20 (−13.96 – 7.56)	0.555	–	–
Constant		76.34 (60.44 – 92.24)	< 0.001	76.69 (63.78 – 89.6)	< 0.001

**Table 3:** Results of multiple linear regression model for explicit dismay attitude.

		Initial model		Final model	
		Coefficient (95%CI)	p	Coefficient (95%CI)	p
Knowledge score					
	Epidemiological	−0.08 (−0.23 – 0.07)	0.315	–	–
	Diagnosis	0.25 (−0.01 – 0.50)	0.058	–	–
	Treatment	0.04 (−0.09 – 0.16)	0.571	–	–
Workload (hours/week)		−0.48 (−0.8 – −0.15)	0.005	−0.49 (−0.80 – −0.18)	0.003
Good basis on cognitive alterations in older adults during medical school (ref. = Did not have)					
	Yes, I had	−6.64 (−15.2 – 1.92)	0.126	–	–
	I cannot remember	−13.88 (−29.6 – 1.84)	0.083	–	–
Took, at some time in life, courses on cognitive alteration in older adults		−7.31 (−18.12 – 3.49)	0.182	–	–
Constant		56.47 (42.31 – 70.63)	< 0.001	58.32 (47.68 – 68.97)	< 0.001

For the optimistic delegating attitude score ( Table 2 ), the predictor variables considered were “ever performing courses on cognitive alteration in older adults,” “having medical residency or equivalent specialization” and “workload” (significant at 20%), in addition to the scores of the three subscales of knowledge. The scores of diagnostic knowledge (p = 0.037), workload (p = 0.002) and having completed residency/specialization (p = 0.033) remained significant in the final model. Thus, the higher the score of diagnostic knowledge or workload, the lower the physician's optimistic delegating attitude score (0.25 points and 0.53 points less, respectively, each increase of 1 point in the diagnostic knowledge score and 1 more hour of work). In addition, physicians with residency or completed specialization have 9.5 points less, on average, in the score of optimistic delegating attitude.

For the attitude of explicit dismay score ( Table 3 ), taking courses on cognitive impairment in older adults, having received a good basis of cognitive alterations in older adults during medical school and workload (significant to 20%) were considered as predictor variables, in addition to knowledge scores. Only workload (p = 0.003) remained significant in the final model. Thus, with each 1-hour increase in the weekly working day, there was a reduction of 0.5 points in the attitude of explicit dismay score.

## DISCUSSION

The knowledge quiz and the attitude quiz, initially created for the evaluation of general practitioners working in the United Kingdom ^1^ , and already cross-culturally adapted for Brazil ^5^ , proved to be extremely useful for the assessment of Brazilian general practitioners working in basic health units. Half of the physicians interviewed had five years of education, which shows the high turnover of this group in primary care; only one third worked exclusively in primary care, and most did not have and did not attend any specialization or medical residency. When assessing the level of knowledge, all indexes were low, and the worst of them was the diagnosis level, which reached a correction level of 39.5%. Turner's original study ^1^ , conducted in Scotland with general practitioners in 2004, showed diagnostic correct rates of 74%. A study conducted with physicians attending residency in a university in São Paulo ^6^ , with the application of the same instruments, showed a diagnostic correct rate of 58.7%.

The variables related to having taken courses during medical school, or at some time in life as a professional update, and having attending medical residency or equivalent proved to be very important in the relationship with knowledge about dementia for professional practice. The study showed that obtaining more knowledge by the general practitioner – for example, taking courses on cognitive alterations – positively impacted their knowledge compared with professional who had never been updated on the subject. The workload was also an impact factor, and the longer graduation time may have meant greater experience.

The four patterns of attitudes identified show a spectrum that varies from a frankly optimistic and proactive posture towards the patient to a frankly pessimistic posture, which does not acknowledge benefits in the diagnosis and treatment of patients with dementia. The graduation time and having attended medical residency or specialization are factors that contributed to a more optimistic attitude of the doctor towards patients with dementia. The level of knowledge of the physician was often positively correlated with more favorable and receptive attitudes in the care of these patients.

The attitude profile of Brazilian general practitioners is different from that of British professionals ^1^ , possibly due to marked cultural differences. Some examples: in Britain, physicians agree almost 100% with the statement that much can be done for the quality of life of patients with dementia and their healthcare, while in this study some doctors still have doubts about it. Another aspect is the role of primary care and specialized services in the diagnosis of dementia – while in this study 56% of physicians considered that the diagnosis should be made in a specialized service, in the United Kingdom, this rate was 33%, showing that the generalists there, back in 2004, already saw greater possibilities for primary care to take in these services than the doctors in São Paulo. Due to these differences, it was not unexpected that the factor analysis of attitudes obtained from the perceptions of physicians in São Paulo was substantially different from that of British physicians. Conceptually, the analyses look similar. There is a profile of more optimistic (in Turner's study ^1^ , classified as a “heartfelt” factor, and in this study, factors of proactive optimism and delegating optimism) and pessimistic attitudes (“heartsink,” for Turner ^1^ , and implicit and explicit dismay factors in São Paulo), but in practice the items were not distributed accordingly. Of the four items that make up the “heartsink” factor, in this study an item is in the proactive optimism factor (“the primary care team has a very limited role in the care of people with dementia”), and of the three items of the “heartfelt” factor, in this study an item is in the implicit dismay factor (“families prefer to be informed about their relative's dementia as soon as possible”).

The factor that showed the highest reliability is the attitude of proactive optimism, despite the low variability with the data studied. Regarding the optimistic delegating attitude factor, we perceived that, although it is still positive, it is an attitude of the physician to take no responsibility for the management of the problems presented. This attitude was less used by physicians who spent more time working in the BHU during the week, they knew how to identify the diagnosis of dementia and had attended residency or equivalent degree.

The attitude of implicit dismay proved to be a weak factor from the point of view of factor analysis, because reliability was very low. Finally, the pattern of attitude of explicit dismay showed that working time in the BHU was, again, the variable of greater interference on attitude, that is, the less time to work in the BHU had the doctors, the greater the chance of them having a pessimistic attitude, of not recognizing the importance of the diagnosis of dementia in older adults.

The association between greater experience of the general practitioner and increase in positive attitudes, verified by Turner ^1^ and Ólafsdóttir ^7^ , was also observed in our study, in which there was an association between medical residency/specialization and more positive attitudes, as a lower delegation of care of patients with dementia to other services. In the study by Jacinto ^6^ , there was no association between experience and positive attitudes, since undergraduates showed more positive attitudes than resident physicians in the early stages.

In this study, variables such as course on cognitive alterations, longer graduation time, having attended medical residency or specialization positively impacted the better performance of general practitioners on the scale of knowledge and attitudes.

To improve the care of patients with dementia in primary care in the city of São Paulo, it is necessary to invest in a medical training that includes the performance of medical residency or specialization, as well as courses on cognitive alterations. General practitioners who have good knowledge about dementia associated with optimistic attitudes are expected to have the greatest success in the recognition, diagnosis and management of these patients.

One of the limitations of this study was the small number of the sample. Another factor is the difficulty of comparing the results with other studies from abroad, since the culture, the medical training and the definition of general practitioner are quite different ^8^^–^^10^ .

## Footnotes

a The alpha coefficient typically ranges from 0 to 1. However, in reality, there is no lower limit for this coefficient. The closer to 1, the greater the consistency between items on a scale or subscale. George and Mallery (George D, Mallery P. 2003, SPSS for Windows step by step: A simple guide and reference, 11.0 update – 4^th^. Ed. Boston: Allyn & Bacon) provide some rules: 0.9 — excellent; from 0.8 to 0.9 inclusive — good; from 0.7 to 0.8 inclusive — acceptable; from 0.5 to 0.7 inclusive – poor; and below 0.5 – unacceptable.
