# Supplementary material for: Older adults with dementia: knowledge and attitudes of physicians in health units
Source: Rev Saude Publica. 2020 Nov 27;54:137. doi: 10.11606/s1518-8787.2020054002451 (PMC7703527; doi:10.11606/s1518-8787.2020054002451)
Supplement: Translation

# Translation: Idosos com demência: conhecimentos e atitudes dos médicos nas unidades de saúde

**Authors:** Ana Beatriz Quintes Steiner, Alessandro Ferrari Jacinto, Vanessa de Albuquerque Citero

**Affiliations:** I São PauloSP Brasil Universidade Federal de São Paulo. Escola Paulista de Medicina. Programa de Pós-Graduação em Psiquiatria e Psicologia Médica. São Paulo, SP, Brasil; II São PauloSP Brasil Universidade Federal de São Paulo. Escola Paulista de Medicina. Departamento de Medicina. São Paulo, SP, Brasil; III São PauloSP Brasil Universidade Federal de São Paulo. Escola Paulista de Medicina. Departamento de Psiquiatria. São Paulo, SP, Brasil

**Keywords:** Médicos de Atenção Primária, Conhecimentos, Atitudes e Prática em Saúde, Pacientes, Idoso, Demência

## Abstract

**OBJETIVO:**

Descrever os conhecimentos e atitudes dos médicos generalistas da rede básica de saúde da cidade de São Paulo em relação a pacientes com demência e identificar padrões de atitudes.

**MÉTODOS:**

Foram sorteadas aleatoriamente 10% das unidades básicas de saúde da cidade de São Paulo (n = 45), distribuídas proporcionalmente em seis coordenadorias regionais de saúde. Foram entrevistados até dois médicos generalistas em cada unidade, obtendo-se ao todo 81 médicos entrevistados. Eles responderam à versão traduzida e adaptada transculturalmente para o Brasil de dois questionários britânicos, o *knowledge quiz* (conhecimentos sobre demências) e o *attitude quiz* (atitudes frente ao paciente dementado), além de um questionário sociodemográfico e ocupacional para o entendimento do perfil dos médicos generalistas que atuam na atenção primária. Realizaram-se análise descritiva dos dados, análise fatorial dos principais componentes do *attitude quiz* e estudo de associação entre atitudes e conhecimentos, além do teste de regressão linear múltipla para determinar a relação entre o perfil ocupacional e de conhecimento em demência sobre os padrões de atitudes.

**RESULTADOS:**

Os médicos entrevistados tinham mediana de tempo de formado de cinco anos; 35,8% trabalhavam exclusivamente com atenção primária, e menos de 40% tinham concluído, ou estavam cursando, residência médica ou especialização. Os médicos mostraram um conhecimento menor sobre diagnóstico de demência do que sobre a epidemiologia da doença e seu manejo terapêutico. Suas atitudes em relação a pacientes dementados resultaram em quatro fatores: otimismo proativo, otimismo delegador, desalento implícito e desalento explícito. O estudo de regressão mostrou que a atitude de desalento explícito diminui quanto maior for a jornada de trabalho semanal do médico nas unidades, e que a atitude otimista delegadora do médico diminui na mesma situação.

**CONCLUSÕES:**

O investimento em capacitação é fundamental para melhorar o desempenho do médico no campo da demência na atenção primária.

## INTRODUÇÃO

Quadros demenciais têm importante impacto no indivíduo portador, em sua família, na saúde pública, na sociedade e na economia ^1^^,^^2^ . Identificar precocemente indivíduos no início do quadro demencial pode ser uma maneira de intervir na progressão da doença, e médicos generalistas de unidades básicas de saúde (UBS) devem estar preparados para esta realidade.

O termo “médico generalista” no Brasil é muitas vezes utilizado de forma indistinta. Segundo o Cremesp (2010) ^3^ , o termo deve ser apenas dirigido a médicos egressos da faculdade de medicina que não fizeram residência médica ou especialização reconhecida pela Associação Médica Brasileira.

Analisar as atitudes de médicos generalistas frente a pacientes dementados é importante para obter informações sobre o cuidado que esses pacientes recebem na atenção primária. Para isso, deve-se compreender o nível de conhecimento dos médicos sobre o tema e que fatores influenciam suas atitudes. O objetivo deste estudo é avaliar a relação entre padrões de atitudes de médicos generalistas frente ao paciente idoso com demência e seu nível de conhecimento.

## MÉTODOS

Este estudo foi aprovado pelo Comitê de Ética da Escola Paulista de Medicina (nº 1.113.179) e pelo Comitê de Ética da Secretaria Municipal da Prefeitura de São Paulo (nº 1.076.944).

Foi realizado estudo transversal descritivo. A coleta de dados ocorreu em 2017, quando a cidade de São Paulo possuía 451 UBS, distribuídas em seis coordenadorias regionais de saúde (2019) ^4^ (Norte = 88, Sul = 121, Centro = 9, Oeste = 29, Leste = 112 e Sudeste = 92).

Estima-se que cada UBS tenha dois médicos generalistas. Dessa forma, para a coleta de dados, foram sorteadas aleatoriamente, compondo uma amostra randômica, 10% das UBS da cidade de São Paulo (N = 45), distribuídas proporcionalmente nas seis regiões da cidade (Norte = 9, Sul = 12, Centro = 1, Oeste = 3, Leste = 11 e Sudeste = 9). Foram entrevistados, quando possível, dois médicos por UBS, totalizando uma amostra estimada de 90 médicos. No caso da UBS sorteada ter apenas um médico, foi sorteado mais uma UBS da mesma região para completar a amostragem. Dessa forma, foram abordadas 48 UBS ( Figura 1 ), e 81 médicos concordaram em participar da pesquisa. O critério de inclusão do médico foi trabalhar nas UBS como generalista, independentemente da formação, sem critérios de exclusão adicionais.

**Figura:** Organograma da coleta de dados.

Uma vez sorteadas as unidades, o entrevistador entrou em contato com as coordenadorias de saúde de cada região. Com a permissão e a liberação da pesquisa pelos coordenadores, os gerentes de cada UBS foram contatados por telefone ou *e-mail* . Inicialmente, as entrevistas para aplicação dos questionários foram agendadas de acordo com o melhor dia e horário para a unidade. O entrevistador foi pessoalmente a 34 UBS. Na ocasião das visitas, os dois médicos de cada unidade, após preenchimento do termo de consentimento livre e esclarecido, responderam sozinhos os questionários. Nas unidades restantes (14), por solicitação dos gerentes das UBS, a pesquisa foi enviada por *e-mail* . Respondidas, as pesquisas foram reenviadas ao pesquisador. Não foram observadas diferenças na qualidade do preenchimento dos questionários entre os que foram realizados pessoalmente e os que foram entregues por *e-mail* .

Foi aplicada a versão traduzida e adaptada transculturalmente de dois questionários, um sobre o conhecimento do médico a respeito da demência e outro sobre sua atitude em relação a esses pacientes ^1^^,^^5^ , além do questionário sociodemográfico e profissional do médico, desenvolvido para esta pesquisa.

- Questionário de conhecimentos ( *knowledge quiz* ): traduzido e adaptado transculturalmente para o Brasil sobre conhecimentos do médico generalista a respeito de alterações cognitivas ^5^ . São 14 testes de múltipla escolha, com cinco alternativas possíveis, sendo uma das alternativas “eu não sei”, com apenas uma resposta correta. O questionário é dividido em três domínios, que abrangem conhecimentos relacionados a fatores epidemiológicos (4 questões), de diagnóstico (7) e de manejo de pacientes com demência (3). O questionário já vem sendo aplicado em médicos generalistas do Reino Unido há algum tempo ^1^ .
- Questionário de atitudes frente ao idoso com demência ( *attitude quiz* ): traduzido e adaptado transculturalmente para o Brasil sobre atitudes frente ao paciente dementado ^5^ . A análise fatorial do instrumento em inglês foi coincidente com o a do documento em português. São dez afirmações relacionadas a atitudes e opiniões de médicos generalistas frente ao manejo e prognóstico de demências. Utilizando uma escala Likert de cinco pontos, que variam entre “concordo plenamente” e “discordo plenamente”, os médicos generalistas descrevem como lidam com o atendimento de pacientes dementados. Na versão britânica ^1^ , as dez afirmações compõem dois fatores de atitudes, uma mais positiva e outra mais negativa, no manejo do médico com o idoso que tem a doença. A análise deste instrumento implicou a conversão do escore de cada item para a variação de 0 a 100, e considerou a reversão das respostas das afirmações 6, 7, 8, 9 e 10.
- Questionário sociodemográfico e ocupacional do médico: informações como idade do médico, tempo de formação, carga horária na atenção básica, ter realizado residência médica/especialização e ter participado alguma vez de cursos sobre alterações cognitivas são importantes para a descrição da amostra.

De início, os dados foram analisados descritivamente. Para as variáveis categóricas, foram apresentadas frequências absolutas e relativas, e para as variáveis numéricas, medidas-resumo (média, quartis, mínimo, máximo e desvio-padrão).

A existência de associações entre duas variáveis categóricas foi verificada utilizando-se o teste de qui-quadrado, ou alternativamente, em casos de amostras pequenas, o teste exato de Fisher. Havendo diferenças na distribuição, utilizou-se o resíduo ajustado padronizado para identificar as diferenças locais – caselas com valores absolutos acima de 1,96 evidenciam associações (locais) entre as categorias relativas a essas caselas.

A associação linear entre duas variáveis numéricas foi avaliada via correlação de Pearson (r^P^) ou Spearman (r^S^).

Foi utilizado o teste *t* de Student e a análise de variância (Anova) para comparar as médias entre dois e três grupos, respectivamente. Ambos apresentam como pressupostos a normalidade na distribuição dos dados e a homocedasticidade, as quais foram verificadas pelo teste de Kolmogorov-Smirnov e pelo teste de Levene, respectivamente. Em caso de violação da suposição de homocedasticidade, os graus de liberdade da estatística foram corrigidos utilizando-se a correção de Brown-Forsythe. Em caso de violação da suposição de normalidade na Anova, empregou-se alternativamente o teste não paramétrico de Kruskal-Wallis. As médias dos escores de conhecimento foram comparadas pelo teste não paramétrico de Friedman. Havendo diferenças de médias na Anova, os grupos distintos foram identificados pelo teste de Duncan, para se manter o nível global de significância. Já para o teste de Kruskal-Wallis ou teste de Friedman, foram empregadas as comparações múltiplas Dunn-Bonferroni.

Para avaliar a dimensionalidade da escala de atitude composta por dez itens, realizou-se análise fatorial exploratória (AFE) pelo método por componentes principais e rotação ortogonal Varimax. O critério para a seleção do número de fatores foi de autovalores acima do valor 1. Foram apresentados o coeficiente de adequação da amostra de Kaiser-Meyer-Olkin (KMO) e o teste de esfericidade Bartlett, que avalia a significância global de todas as correlações entre os itens da escala considerados. Em seguida, foram calculados os coeficientes alfa de Cronbach, para avaliar a consistência interna dos itens que compunham cada fator.

Para avaliar os efeitos das características de formação e profissional dos médicos, bem como dos escores de conhecimento e de atitudes, empregou-se o modelo de regressão linear múltipla, o qual também apresenta como um dos pressupostos a normalidade nos dados. Inicialmente, foram incluídas no modelo todas as variáveis explicativas significantes a 20% na análise univariada. Em seguida, as variáveis não significantes a 5% foram excluídas uma a uma, por ordem de significância (método *backward* ).

Para todos os testes estatísticos, foi adotado um nível de significância de 5%. As análises foram realizadas utilizando-se o pacote estatístico SPSS 20.0.

## RESULTADOS

Verificou-se que 54,3% dos médicos eram do sexo masculino, procedentes da Grande São Paulo, 35,8% trabalhavam exclusivamente na prefeitura de SP, em atenção básica, e 98,8% trabalhavam como clínicos gerais na UBS. Adicionalmente, 54,3% apontaram ter adquirido, durante a graduação, boa base sobre alterações cognitivas (demências) em idosos, e 18,5% realizaram, alguma vez na vida, cursos específicos sobre a doença. Além disso, 25,9% já realizaram residência médica ou especialização equivalente, e 19,8%, pós-graduação senso estrito.

Em média, os médicos possuíam 10 anos de formado (DP = 11,8 anos), sendo observado um tempo mínimo de 1 ano e máximo de 45 anos. Com relação à carga horária na UBS, os médicos trabalhavam, em média, 31,7 horas (DP = 12,3 horas).

As médias dos conhecimentos por domínios não foram similares entre si (p < 0,001). Já as médias de conhecimento epidemiológico e de manejo foram similares, e ambas superiores às de diagnóstico.

Encontraram-se diferenças de médias do escore de conhecimento apenas por realização, alguma vez na vida, de cursos sobre alteração cognitiva em idosos (p < 0,001); isto é, aqueles que realizaram cursos apresentaram média de conhecimento total superior à daqueles que não fizeram curso. Este efeito se manteve quando foi avaliado apenas o conhecimento em relação à epidemiologia (p = 0,027), mas não quando foi avaliado o conhecimento em relação ao diagnóstico.

Observou-se correlação positiva entre carga horária realizada na UBS e escore total de conhecimento (r^S^= 0,244, p = 0,028) e de diagnóstico (r^S^= 0,295, p = 0,007). Notou-se também fraca correlação positiva entre tempo de formado e escore de manejo (r^S^= 0,237, p = 0,033). Já a correlação entre porcentagem de idosos atendidos na unidade de saúde e escore de conhecimento de diagnóstico mostrou-se negativa (r^S^= −0,319, p = 0,021).

Foi realizada análise fatorial exploratória para avaliar a dimensionalidade da escala de atitude sugerida pelos dados ( Tabela 1 ). Obtiveram-se quatro fatores, que, juntos, explicam 63,2% da variabilidade total dos dez itens. Os resultados da análise fatorial podem ser interpretados pelas “cargas fatoriais”, indicando os itens que compõem cada fator. Na observação do conjunto de item por fator, destacaram-se as seguintes qualidades:

- Fator 1 – atitude otimista proativa: valores altos deste indicador apontam a percepção de médicos de que podem contribuir para melhorar a qualidade do cuidado do idoso com demência e que eles estão dispostos a efetivar esse cuidado;
- Fator 2 – atitude otimista delegadora: valores altos deste indicador apontam que os médicos têm a percepção de que o manejo do idoso com demência pode ser feita com otimismo, porém eles não se responsabilizam por isso e delegam a outras instâncias;
- Fator 3 – atitude de desalento implícito: valores altos deste indicador apontam que os médicos não se percebem pessimistas no cuidado do idoso, mas depositam no familiar a dificuldade de ver futuro no manejo do idoso com demência, como se estivessem desalentados por procuração;
- Fator 4 – atitude de desalento explícito: valores altos deste indicador apontam que os médicos têm a percepção clara de que será infrutífero qualquer manejo deles com o idoso com demência.

**Tabela 1:** Cargas fatoriais, autovalores, porcentagem da variância explicada e coeficiente alfa de Cronbach dos quatro fatores da escala.

Itens	Atitudes				Comunalidades *
	FATOR 1 Otimista proativo	FATOR 2 Otimista delegador	FATOR 3 Desalento implícito	FATOR 4 Desalento explícito	
3. Muito pode ser feito para melhorar a qualidade de vida de pessoas com demência	0,756	0,123	−0,100	−0,190	0,632
1. Muito pode ser feito para melhorar a qualidade de vida de cuidadores de pessoas com demência	0,718	−0,039	−0,375	0,154	0,682
4. Fornecer diagnóstico geralmente é mais útil do que prejudicial	0,701	0,091	0,228	−0,004	0,552
5. A demência é mais bem diagnosticada em serviços especializados	0,218	0,717	−0,285	−0,025	0,643
10. A equipe de atenção primária tem papel muito limitado no cuidado de pessoas com demência	0,058	0,684	0,181	0,076	0,509
7. É melhor conversar com o paciente utilizando eufemismos	−0,146	0,604	0,410	0,129	0,572
9. Não vale a pena direcionar as famílias para serviços especializados quando elas não querem usá-los	−0,164	0,207	0,732	0,100	0,616
2. As famílias preferem ser informadas sobre a demência de seu parente o mais rápido possível	0,499	−0,147	0,647	−0,081	0,696
6. Pacientes com demência podem esgotar recursos com resultado pouco positivo	0,144	−0,054	0,060	0,892	0,823
8. Tratar a demência costuma ser mais frustrante do que gratificante	−0,293	0,250	−0,001	0,671	0,599
Autovalores	2,03	1,50	1,44	1,35	
Porcentagem (%) da variância total explicada	20,34	15,01	14,43	13,46	
Porcentagem acumulada (%) da variância total explicada	20,34	35,35	49,78	63,24	
Alfa de Cronbach	0,629	0,468	0,255	0,470	

KMO = 0,534; Teste de esfericidade de Bartlett-Chi (45) = 119,47 (p < 0,001).

* A análise fatorial permite a decomposição da variância de cada item em duas partes: parte comum e parte específica. A parcela da variância comum – devida a fatores comuns – é denominada comunalidade. Valores inferiores a 0,50 indicam que os itens correspondentes estão pouco representados na análise fatorial.

Verificaram-se, para todos os fatores, consistências internas inaceitáveis segundo os coeficientes alfa de Cronbach ^a^ , exceto para o fator de atitude otimista proativa, que se mostrou adequado (alfa de Cronbach = 0,629). Os escores de cada fator foram gerados como soma dos valores atribuídos a cada item na forma revertida e re-escalonados de tal modo que variassem de 0 a 100.

Não foram encontradas diferenças de médias do escore de atitude otimista proativa, de atitude otimista delegadora e de atitude de desalento explícito em relação a características sociodemográficas e profissionais. No entanto, quanto à atitude de desalento implícito, verificaram-se diferenças de médias do escore com “ter boa base sobre alterações cognitivas em idosos durante a graduação em medicina” (p = 0,010). Dessa forma, aqueles que apontaram ter recebido na graduação boa base sobre a doença apresentaram média de escore de atitude de desalento implícito superior à dos que citaram não ter tido boa base.

Observou-se correlação negativa entre carga horária e escore de atitude otimista proativa (r^P^= −0,375, p = 0,001) e de desalento explícito (r^P^= −0,331, p = 0,003). Já a correlação entre porcentagem de idosos atendidos na UBS e o escore de atitude de desalento implícito mostrou-se positiva (r^P^= 0,277, p = 0,046), mas não houve correlação deste dado com a atitude otimista delegadora.

Encontraram-se correlações negativas entre atitude otimista delegadora e escore de conhecimento total (r^S^= −0,225, p = 0,044) e de diagnóstico (r^S^= −0,305, p = 0,006). Adicionalmente, verificaram-se correlações positivas entre atitude de desalento implícito e escore de conhecimento total (r^S^= 0,257, p = 0,021), epidemiológico (r^S^= 0,224, p = 0,044) e de manejo (r^S^= 0,341, p = 0,002).

Para avaliar os efeitos das características profissionais dos médicos, bem como dos escores de conhecimento e de atitude, foram ajustados modelos de regressão linear múltipla para os escores de atitude otimista delegadora ( Tabela 2 ) e de desalento explícito ( Tabela 3 ). Não foram ajustados modelos para atitude de otimismo proativo, por não apresentar associação com nenhuma das características dos médicos e escores de conhecimento. Com relação à atitude de desalento implícito, não foram ajustados modelos devido à baixa consistência interna (α = 0,255).

**Tabela 2:** Modelo de regressão linear múltipla para atitude otimista delegadora.

		Modelo inicial		Modelo final	
		Coeficiente (IC95%)	p	Coeficiente (IC95%)	p
Escore de conhecimento					
	Epidemiológico	−0,04 (−0,19 – 0,12)	0,628	–	–
	Diagnóstico	−0,24 (−0,50 – 0,02)	0,075	−0,25 (−0,49 – −0,02)	0,037
	Tratamento	0,03 (−0,10 – 0,15)	0,665	–	–
Carga horária (horas/semana)		−0,51 (−0,87 – −0,16)	0,005	−0,53 (−0,86 – −0,21)	0,002
Fez residência/especialização (ref. = não)					
	Sim, concluído	−9,37 (−18,75 – 0,01)	0,050	−9,52 (−18,24 – −0,81)	0,033
	Está cursando	1,18 (−11,38 – 13,74)	0,852	–	–
Realização, alguma vez na vida, de curso sobre alteração cognitiva em idosos		−3,20 (−13,96 – 7,56)	0,555	–	–
Constante		76,34 (60,44 – 92,24)	< 0,001	76,69 (63,78 – 89,6)	< 0,001

**Tabela 3:** Resultados do modelo de regressão linear múltipla para atitude de desalento explícito.

		Modelo inicial		Modelo final	
		Coeficiente (IC95%)	p	Coeficiente (IC95%)	p
Escore de conhecimento					
	Epidemiológico	−0,08 (−0,23 – 0,07)	0,315	–	–
	Diagnóstico	0,25 (−0,01 – 0,50)	0,058	–	–
	Tratamento	0,04 (−0,09 – 0,16)	0,571	–	–
Carga horária (horas/semana)		−0,48 (−0,8 – −0,15)	0,005	−0,49 (−0,80 – −0,18)	0,003
Boa base sobre alterações cognitivas em idosos durante a sua graduação (ref. = Não teve)					
	Sim, teve	−6,64 (−15,2 – 1,92)	0,126	–	–
	Não lembro	−13,88 (−29,6 – 1,84)	0,083	–	–
Realização, alguma vez na vida, de curso sobre alteração cognitiva em idosos		−7,31 (−18,12 – 3,49)	0,182	–	–
Constante		56,47 (42,31 – 70,63)	< 0,001	58,32 (47,68 – 68,97)	< 0,001

Para o escore de atitude otimista delegadora ( Tabela 2 ), foram consideradas como variáveis preditoras “realização alguma vez na vida de cursos sobre alteração cognitiva em idosos”, “ter residência médica ou especialização equivalente” e “carga horária” (significantes a 20%), além dos escores das três subescalas de conhecimento. Permaneceram significantes no modelo final o escore de conhecimento de diagnóstico (p = 0,037), carga horária (p = 0,002) e ter residência/especialização concluída (p = 0,033). Dessa forma, quanto maior é o escore de conhecimento de diagnóstico ou carga horária de trabalho, menor é o escore de atitude otimista delegadora do médico (0,25 pontos e 0,53 pontos a menos, respectivamente, a cada aumento de 1 ponto no escore de conhecimento de diagnóstico e 1 hora a mais de trabalho). Além disso, médicos com residência ou especialização concluída têm 9,5 pontos a menos, em média, no escore de atitude otimista delegadora.

Para o escore de atitude de desalento explícito ( Tabela 3 ), foram consideradas como variáveis preditoras a realização, alguma vez na vida, de cursos sobre alteração cognitiva em idosos, ter recebido durante a graduação boa base sobre alterações cognitivas em idosos e carga horária (significante a 20%), além dos escores de conhecimento. Permaneceu significante no modelo final apenas a carga horária (p = 0,003). Dessa forma, a cada aumento de 1 hora na jornada de trabalho semanal, ocorreu redução de 0,5 pontos no escore de atitude de desalento explícito.

## DISCUSSÃO

O *knowledge quiz* e o *attitude quiz,* criados inicialmente para avaliação de médicos generalistas atuantes no Reino Unido ^1^ , e já adaptados transculturalmente para uso no Brasil ^5^ , mostraram-se extremamente úteis para a avaliação de médicos generalistas brasileiros atuantes nas unidades básicas de saúde. Metade dos médicos entrevistados tinha cinco anos de formados, o que mostra a alta rotatividade deste grupo na atenção primária; apenas um terço trabalhava exclusivamente na atenção primária, e a maioria não tinha e não fazia nenhuma especialização ou residência médica. Ao avaliar o nível de conhecimento, todos os índices mostraram-se baixos, e o pior deles foi o de diagnóstico, que chegava ao nível de acerto de 39,5%. O estudo original de Turner ^1^ , realizado na Escócia com médicos generalistas em 2004, evidenciou índices de acerto de diagnóstico de 74%. Estudo realizado com médicos residentes de uma universidade em São Paulo ^6^ , com a aplicação dos mesmos instrumentos, mostrou porcentagem de acerto de diagnóstico de 58,7%.

As variáveis relacionadas a ter feito cursos durante a graduação, ou alguma vez na vida como atualização profissional, e ter feito residência médica ou equivalente mostraram-se muito importantes na relação com o conhecimento sobre demência para a prática profissional. O estudo mostrou que a obtenção de mais conhecimento pelo médico generalista – por exemplo, realizando cursos sobre alterações cognitivas – impactava positivamente em seu conhecimento em comparação com o profissional que nunca se atualizou sobre o assunto. A carga horária também foi fator de impacto, e o maior tempo de formado pode ter significado maior experiência.

Os quatro padrões de atitudes identificados demonstram um espectro que varia de uma postura francamente otimista e proativa diante do paciente a uma postura francamente pessimista, que não reconhece benefícios no diagnóstico e tratamento de pacientes com demência. O tempo de formado e ter feito residência médica ou especialização são fatores que contribuíram para uma atitude mais otimista do médico em relação a pacientes portadores de demência. O nível de conhecimento do médico muitas vezes correlacionou-se positivamente com atitudes mais favoráveis e receptivas no cuidado com esses pacientes.

O perfil de atitudes dos médicos generalistas brasileiros é distinto daquele dos profissionais britânicos ^1^ , possivelmente por diferenças culturais marcantes. Alguns exemplos: na Grã-Bretanha, médicos registram quase absoluto 100% de concordância com a afirmação de que muito pode ser feito pela qualidade de vida do paciente com demência e dos cuidados para com ele, enquanto no presente estudo ainda há médicos que demonstram duvidas em relação a isso. Outro aspecto é o papel da atenção primária e dos serviços especializados no diagnóstico da demência – enquanto no presente estudo 56% dos médicos consideraram que o diagnóstico deve ser feito em serviço especializado, no Reino Unido esta taxa foi de 33%, mostrando que os generalistas de lá, em 2004, já viam maiores possibilidades de a atenção primária cuidar desses serviços do que os médicos de São Paulo. Devido a essas diferenças, não foi inesperado que a análise fatorial das atitudes obtida a partir das percepções dos médicos de São Paulo tenha sido substancialmente diferente daquela dos médicos britânicos. Conceitualmente, as análises parecem semelhantes. Há um perfil de atitudes mais otimistas (no estudo de Turner ^1^ , classificada como fator “ *heartfelt* ”, e no presente estudo, fatores de otimismo proativo e otimismo delegador) e mais pessimistas (“ *heartsink* ”, para Turner ^1^ , e fatores de desalento implícito e explícito em São Paulo), mas na prática os itens não foram distribuídos de forma correspondente. Dos quatro itens que compõem o fator “ *heartsink* ”, no presente estudo um item está no fator de otimismo proativo (“a equipe de atenção primária tem papel muito limitado no cuidado de pessoas com demência”), e dos três itens do fator “ *heartfelt* ”, no presente estudo um item está no fator de desalento implícito (“as famílias preferem ser informadas a respeito da demência de seu parente o mais rápido possível”).

O fator que mostrou maior confiabilidade é a atitude de otimismo proativo, apesar da baixa variabilidade com os dados estudados. Em relação ao fator de atitude otimista delegadora, percebe-se que, apesar de ainda ser positiva, é uma atitude do médico para afastar de si a responsabilidade pelo manejo dos problemas apresentados. Essa atitude foi menos utilizada por médicos que ficavam mais tempo trabalhando na UBS durante a semana, sabiam identificar o diagnóstico de demência e tinham realizado residência ou equivalente.

A atitude de desalento implícito mostrou-se um fator fraco do ponto de vista da análise fatorial, pois a confiabilidade foi muito baixa. Por fim, o padrão de atitude de desalento explícito mostrou que o tempo de trabalho na UBS foi, novamente, a variável de maior interferência sobre a atitude, isto é, quanto menos tempo para trabalhar na UBS tinha o médico, maior era a chance de ele ter uma atitude pessimista, de não reconhecimento da importância do diagnóstico de demência no idoso.

A associação entre maior experiência do médico generalista e aumento de atitudes positivas, verificada por Turner ^1^ e Ólafsdóttir ^7^ , também foi observada no presente estudo, no qual se verificou associação entre residência médica/especialização e atitudes mais positivas, como menor delegação do cuidado do paciente dementado a outros serviços. No estudo de Jacinto ^6^ , não se encontrou associação entre experiência e atitudes positivas, uma vez que graduandos mostraram atitudes mais positivas do que médicos residentes nas fases iniciais.

No presente estudo, variáveis como realização de curso sobre alterações cognitivas, maior tempo de formado, ter feito residência médica ou especialização impactaram positivamente no melhor desempenho dos médicos generalistas na escala de conhecimentos e atitudes.

Para melhorar o atendimento a pacientes portadores de demência na atenção básica na cidade de São Paulo, é preciso investir em uma formação médica que inclua a realização de residência médica ou especialização, bem como cursos sobre alterações cognitivas. Imagina-se que médicos generalistas que apresentem bom conhecimento sobre demência associado a atitudes otimistas são os que terão maior sucesso no reconhecimento, diagnóstico e manejo desses pacientes.

Uma das limitações deste estudo foi o número pequeno da amostra. Outro fator é a dificuldade de comparar os resultados com outros estudos do exterior, uma vez que a cultura, a formação médica e a definição de médico generalista são bastante diferentes. ^8^^–^^10^ .

## Footnotes

a O coeficiente alfa normalmente varia entre 0 e 1. Entretanto, na realidade, não há limite inferior para este coeficiente. Quanto mais próximo de 1, maior será a consistência entre os itens de uma escala ou sub-escala. George e Mallery (George D, Mallery P. 2003, SPSS for Windows step by step: A simple guide and reference, 11.0 update – 4th. Ed. Boston: Allyn & Bacon) fornecem algumas regras: 0,9 – excelente; de 0,8 a 0,9, inclusive – bom; de 0,7 a 0,8, inclusive – aceitável; de 0,5 a 0,7, inclusive – pobre; e abaixo de 0,5 – inaceitável.
